# BMSCs and miR‐124a ameliorated diabetic nephropathy via inhibiting notch signalling pathway

**DOI:** 10.1111/jcmm.13747

**Published:** 2018-07-19

**Authors:** Jiping Sun, Fei Zhao, Wenjing Zhang, Jia Lv, Jing Lv, Aiping Yin

**Affiliations:** ^1^ Department of Nephrology the First Affiliated Hospital of Medical College Xi'an Jiaotong University Xi'an China; ^2^ Department of Obstetrics and Gynecology the First Affiliated Hospital of Medical College Xi'an Jiaotong University Xi'an China

**Keywords:** apoptosis, diabetic nephropathy, kidney injury, miR‐124a, Notch, podocyte

## Abstract

BMSCs are important in replacement therapy of diabetic nephropathy (DN). MiR‐124a exerts effect on the differentiation capability of pancreatic progenitor cells. The objective of this study was to explore the molecular mechanisms, the functions of miR‐124a and bone marrow mesenchymal stem cells (BMSCs) in the treatment of DN. Characterizations of BMSCs were identified using the inverted microscope and flow cytometer. The differentiations of BMSCs were analysed by immunofluorescence assay and DTZ staining. The expression levels of islet cell‐specific transcription factors, apoptosis‐related genes, podocytes‐related genes and Notch signalling components were detected using quantitative real‐time reverse transcription PCR (qRT‐PCR) and Western blot assays. The production of insulin secretion was detected by adopting radioimmunoassay. Cell proliferation and apoptosis abilities were detected by CCK‐8, flow cytometry and TUNEL assays. We found that BMSCs was induced into islet‐like cells and that miR‐124a could promote the BMSCs to differentiate into islet‐like cells. BMSCs in combination with miR‐124a regulated islet cell‐specific transcription factors, apoptosis‐related genes, podocytes‐related genes as well as the activity of Notch signalling pathway. However, BMSCs in combination with miR‐124a relieved renal lesion caused by DN and decreased podocyte apoptosis caused by HG. The protective effect of BMSCs in combination with miR‐124a was closely related to the inactivation of Notch signalling pathway. MSCs in combination with miR‐124a protected kidney tissue from impairment and inhibited nephrocyte apoptosis in DN.

## 1 INTRODUCTION

As one of the most serious complications of diabetes mellitus, diabetic nephropathy (DN) is a serious threat to human health^1^, ^2^. The lack of functional islet cells is a primary cause to the occurrence and development of DN^3^. Studies have found that islet function can be improved by the endogenesis islet cell regeneration. BMSCs have been a key cellular source for replacement therapy of DN in regenerative medicine^4^, ^5^, ^6^, ^7^ and it could be induced to differentiate islet‐like cells^8^, ^9^. BMSCs could migrate into a damaged kidney and regulate immune response. Therefore, the systematic application of BMSCs may improve the function of kidney and remodel the necrotic tissues of DN^10^. In addition, BMSCs can express pancreatic development‐related transcription factors including Pdx‐1, Ngn3, neurogenic differentiation 1 (NeuroD1)^11^. Thus, we speculated that BMSCs could be used to migrate DN through reducing hyperglycaemia and glycosurias. In this study, we aimed to further directionally induce BMSCs to differentiate into islet‐like cell and to explore the role of BMSCs in DN.

MicroRNAs (miRNAs) are a class of endogenous small non‐coding RNA molecules that are composed of 18‐24 nucleotides. By binding to complementary sequences of target messenger RNAs (mRNAs) or non‐coding RNAs^12^, ^13^, ^14^, ^15^, ^16^, miRNAs are important in post‐transcriptional regulation^17^, ^18^. MicroRNA124a (miR‐124a) plays significant roles in pancreatic development, insulin secretion, β cell differentiation, the regulation of blood glucose and lipid metabolism^19^, ^20^, ^21^ In addition, miR‐124a can accurately regulate the development of embryonic pancreas by controlling pancreatic precursor cells counts and regulating the expression levels of pancreatic development‐related transcription factors^20^, ^22^. Studies have shown that the down‐regulation of miR‐124a could reduce the differentiation of pancreatic progenitor cell into β cells^23^, ^24^. Moreover, miRNAs exert important effect on the differentiation of BMSCs into osteoblast, adipocyte and neurocyte^25^, ^26^. However, BMSCs could be detected after one month of transplantation in a DN model. Researchers indicated that the proliferation potency of BMSCs was reduced after the transplantation^27^. Therefore, we speculated that miR‐124a may play an important role in BMSCs differentiation into islet‐like cell.

Notch signalling pathways are a critical signalling pathway in cell differentiation^28^, ^29^, ^30^, ^31^. Studies have demonstrated that the activation of Notch signalling pathway can promote the proliferation of pancreatic precursor cell and the development of exocrine gland^32^, ^33^. Notch signalling pathways also produce critical effects on pancreatic development‐related cells^34^, ^35^. Moreover, the expression levels of Ngn3‐mediated downstream target genes, for example, NeuroD, Hes6‐1 and MyT1, could be down‐regulated by activating Notch signalling pathways^36^. However, the inactivation of Notch signalling pathways can promote the differentiation of endocrine cell in pancreas including α cell and β cell, and reduce insulin resistance^37^, ^38^. Studies have reported that a cross‐talk existed between miRNAs and Notch signalling pathways^39^, ^40^. In our previous study, we found that Notch signalling pathways took part in BMSCs differentiation in vitro. Therefore, we speculated that miR‐124a may play an important role in the differentiation of BMSCs, in which also involves Notch signalling pathways. The combined use of BMSCs and miR‐124a may improve kidney injury in DN rats. The study provides new methods for the prevention and treatment of DN.

## 2 MATERIALS AND METHODS

### 2.1 Isolation, cultivation and identification of BMSCs

Male Sprague‐Dawley (SD) rats aged 6 weeks old (200‐220 g) were provided by the Experimental Animal Center of the First Affiliated Hospital of Medical College, Xi'an Jiaotong University. The experimental animals were treated following the guidelines, and the use and care of laboratory animals was approved by committee of the First Affiliated Hospital of Medical College, Xi'an Jiaotong University. Bilateral femurs of rats were removed and isolated under aseptic conditions. Bone marrow was rinsed with Dulbecco's Minimum Essential Medium (DMEM, Cat No. D1152, Sigma, MO, USA) and centrifuged at 1000 *g* for 10 minutes at 4°C. The supernatant was discarded and then the precipitates were re‐suspended with phosphatic buffer solution (PBS, Cat. No. 28372). Next, 6 mL PBS was gradually dripped into 6 mL of lymphocyte separation medium and centrifuged at 1000 *g* for 10 minutes at 4°C. The cells in the resultant middle white layer were carefully extracted, and washed with PBS. Cells were then plated on a culture dish with DMEM medium (Cat No. D1152, Sigma, MO, USA) containing 15% foetal bovine serum (FBS, cat # SH30071.03) in a humidified atmosphere with 5% CO2 at 37°C. The culture medium was renewed every two days. The growth and morphology of cells were determined using an inverted microscope (Carl Zeiss Microscopy). The cells were sub‐cultured, and the 2‐3 generations of cells were used for FACS analysis. The cells were harvested by incubating with trypsin (cat. no. 0458) and then centrifuged (1000 *g* for 10 minutes at 4°C) to obtain cell pellet. The harvested cells were then washed with ice‐cold PBS once and centrifuged (1500 *g* for 5 minutes at 4°C). The cell pellet was then re‐suspended with 100 μL ice‐cold PBS. Finally, cells were observed under an ordinary optical microscope.

Next, cells were labelled with antibodies against CD29 (Cat: 562154, BD Biosciences, USA), CD44 (Cat: 550974, BD Biosciences, USA) and CD34 (cat# 345801). Cells were then detected using flow cytometer^41^. To be more specific, all antibodies were incubated for 4 minutes in phosphate buffer saline (PBS) with 0.1% of Triton X‐100. For each determination, at least 10 000 cells were analysed using a FACSCalibur cytometer (Becton Dickinson). CellQuest software (BD Biosciences) was used for result analysis. Meanwhile, immunofluorescence assay was performed. In brief, cells were grown in 6‐well plates and fixed in 4% paraformaldehyde for 30 minutes. After incubating with 3% Triton X‐100 for 30 minutes, the slides were blocked with normal serum for 20 minutes and were then incubated with primary antibodies against CD29 (eBioscience, cat. no. 12‐0291), CD44 (cat. no. 338807) and CD34 (eBioscience; cat. no. 12‐0341) overnight. Then the slides were incubated with lgG‐FITC for 2 hours at room temperature, Cells then were observed under a fluorescence microscope (Cat. #IX73, Olympus, Japan).

### 2.2 Differentiation and identification of BMSCs

Differentiation of BMSCs into islet‐like cells in vitro. In brief, cells were cultured in DMEM medium (Cat No. D1152, Sigma, MO, USA) with 5 mmol/L 2‐mercaptoethanol for 24 hours. Cells were incubated into 6‐well plate with Matrigel. Those cells were cultured in DMEM medium (Cat No. D1152, Sigma, MO, USA) with B_27_, 10 μg/L bFGF, 0.1 mmol/L 2‐mercaptoethanol and ITS for 7 days. Next, cells were cultured by DMEM medium with B_27_, 10 μg/L HGF, 20 mmol/L nicotinamide, 0.1 mmol/L 2‐mercaptoethanol and LY294002 for another 7‐21 days. Cells were harvested after 28 days. Cells used for FITC and Cy3 staining were first fixed in 4% paraformaldehyde for 30 minutes after being induced for 24 hours, 7 days, and 14 days. Then, cells were incubated with primary antibodies against nidogen, insulin, glucagon and somatostatin overnight, and then incubated with lgG‐FITC for 2 hours at room temperature. Finally, those cells were observed under a fluorescence microscope (Cat. #IX73, Olympus, Japan). For preparing cells used for dithizone (DTZ) staining^42^, DTZ (Sigma‐Aldrich, Germany) stock solution was first prepared by dissolving 100 mg of DTZ in 5 mL of dimethyl sulfoxide (DMSO, Sigma‐ Aldrich, Germany). Cells induced for 0, 7, 14 and 28 days were washed with PBS buffer twice. Next, 1 mL PBS buffer and 10 μL DTZ stock solutions were added and the cells were stored in an incubator at 37°C for 15 minutes. The crimson‐red‐stained clusters were examined with a phase‐contrast microscope (CKX41, Olympus, Japan).

### 2.3 BMSCs transfection and groups

MiR‐124a mimics, inhibitors and negative control were purchased from GenePharma (Shanghai, China). Then, the mimics, inhibitors or negative control was, respectively, transfected into the cells using HiPerFect Transfection Reagent (Qiagen, Hilden, Germany) following the manufacturer's protocols. As previously reported^27^, BMSCs were harvested 48 hours after the transfection. The final concentration of mimics, inhibitors and negative control was 50 nmol/L. The mimics, inhibitors and negative control were repeatedly transfected every 3 days for long‐term detection. BMSCs were further allocated into five groups: control group (Con; BMSCs with no treatment), induction group (Ind; BMSCs differentiation into islet‐like cells), miR‐124a negative control group (M/Ind; BMSCs were induced for 28 days so as to differentiate into islet‐like cells after transfecting with miR‐124a negative control), miR‐124a mimics group (m/Ind; BMSCs were induced for 28 days so as to differentiated into islet‐like cells after transfecting with miR‐124a mimics), miR‐124a inhibitors group (i/Ind; BMSCs were induced for 28 days so as to differentiated into islet‐like cells after transfecting with miR‐124a inhibitors).

### 2.4 CCK‐8 assay

Cell viability was tested by the CCK8 assay in BMSCs and podocyte. In brief, cells were harvested after being cultured for 28 days, and then were seeded at a density of 4 × 10^3^ cells/well in 96‐well plates and incubated for 0, 12, 24, and 48 hours. Subsequently, 20 μL CCK‐8 was added to each well for other 1‐hour incubation. The optical density (OD) values were read at 570 nm using a microplate reader (Thermo, USA). All experimental concentrations were assessed in triplicate.

### 2.5 Radio immunoassay

The cells in Con, Ind, M/Ind, m/Ind and i/Ind groups were harvested and were used for detecting insulin secretion with radio immunoassay^11^. In brief, cells in all groups were incubated respectively in the DMEM medium containing 2.8 mmol/L glucose, the DMEM medium contains 20 mmol/L glucose and 50 μmol/L IBMX for 2 hours at 37°C. Stimulation index (SI) was calculated and used for testing the sensibility of pancreatic β‐cells to glucose stimulation.

### 2.6 Animal model and groups

Male Sprague‐Dawley (SD) rats aged 6‐8 weeks of age were kept, according to the policy of the local ethics committee of laboratory animal centre, in the Animal Center of the First Affiliated Hospital of Medical College, Xi'an Jiaotong University. Rats were fed with HFD (19.7 kJ/g, 45% fat, Research Diets, Cyagen) for 8 weeks. After fasting for 12 hours, the rats were injected intraperitoneally with low‐dose STZ (100 mg/kg) for 2 weeks to establish DN model. Urine samples over 24 hours were collected. Then venous blood was collected by cutting off rats' tails. Blood glucose, triglyceride, free fatty acid, urinary albumin excretion and creatinine were detected after 8 hours fasting. Rats with a blood glucose level over 16.7 mmol/L were considered as having diabetic, and were therefore accepted in the study^43^. Six groups of rats were used: (i) normal control group (Con, n = 6; the rats were normally fed for 14 weeks), (ii) BMSCs transplanted group (B/Con, n = 6; 3 × 10^6^ BMSCs were injected into rats by tail vein after the rats have been normally fed for 14 weeks), (iii) DN model group (DN, n = 6), (iv) BMSCs transfected with miR‐124a negative control transplanted group (M/DN): 3 × 10^6^ BMSCs transfected with miR‐124a negative control (4 ng/mm^3^) were injected into DN rats by tail vein), (v) BMSCs transfected with miR‐124a mimics transplanted group (m/DN): 3 × 10^6^ BMSCs transfected with miR‐124a mimics (4 ng/mm^3^) were injected into DN rats by tail vein), (vi) BMSCs transfected with miR‐124a inhibitors transplanted group (i/DN): 3 × 10^6^ BMSCs transfected with miR‐124a inhibitors (4 ng/mm^3^) were injected into DN rats by tail vein.

### 2.7 The histological examination of kidney tissue

Samples were isolated and embedded in paraffin to prepare 4‐μm tissue slices. The slices were then investigated using haematoxylin and eosin (HE) staining and Periodic Acid‐Schiff (PAS) staining. As previously described^44^, mesangial expansion index (MEI) was assigned and scored within four levels from 0 to 3. The index scores were defined as the follows: 0, normal glomeruli; 1, matrix expansion occurred in up to 50% of a glomerulus; 2, matrix expansion occurred 50% to 75% of a glomerulus; 3, matrix expansion occurred 75% to 100% of a glomerulus. Scores were assigned for at least 30 glomeruli from kidney slices for each sample, and the means were calculated. Each slide was scored by a pathologist who was unaware of the experimental details.

### 2.8 Cell culture and glucose treatment

A conditionally immortalized murine podocyte cell line was maintained as previously described^45^. Briefly, cells were cultured in RPMI medium 1640 (Sigma‐Aldrich,, St. Louis., MO, USA) supplemented with 10% foetal bovine serum (FBS; Life Technologies, Carlsbad, CA, USA) and 10 units/mL mouse recombinant interferon‐γ (IFN‐γ, R&D Systems, Minneapolis, MN, USA) at 33°C. To induce the differentiation, podocytes were grown in the absence of IFN‐γ for 14 days at 37°C before the experiments and were then stained with synaptopodin. Finally, cells were observed under a fluoroscope microscope.

Podocytes were seeded in a 6‐well plate at a density of 3 × 10^5^ cells/mL with complete culture medium. After 24 hours, the podocytes were exposed to media containing 40 mmol/L of glucose for 24 hours so as to simulate DN environment in vitro (called HG group). Cells were then harvested for further experimental study.

Podocyte was divided into five groups: (i) blank control group (Con; podocyte with no treatment), (ii) HG group, (iii) BMSCs transfected with miR‐124a negative control group (M/HG group: BMSCs were transfected with miR‐124a negative control and the BMSCs with miR‐124a negative control were incubated with podocytes for 24 hours. Then podocytes were exposed to 40 mmol/L glucose for 24 hours) (iv) BMSCs transfected with miR‐124a mimics group (m/HG; podocyte pre‐treated with BMSCs transfected with miR‐124a mimics for 24 hours, and then were exposed to 40 mmol/L glucose for 24 hours), (v) BMSCs transfected with miR‐124a inhibitors group (i/HG; podocyte pre‐treated with BMSCs transfected with miR‐124a inhibitors, and then were exposed to the 40 mmol/L glucose for 24 hours).

### 2.9 Detection of podocyte death

The podocytes of Con, HG, M/HG, m/HG and i/HG groups were trypsinized and fixed with 50% ethanol. The cells were washed three times with cold phosphate‐buffered saline, and then stained with propidium iodide (PI, Invitrogen, cat. no. P3566) for 10 minutes. Cells were finally analysed by a flow cytometry.

### 2.10 Quantitative real‐time reverse transcription PCR (qRT‐PCR)

Total RNA was extracted using RNA Extraction Kit (TIANGEN biochemical science technologies co., Ltd, Beijing, China) following the manufacturer's protocols. In kidney tissues, total RNA was extracted using TRIzol reagent (Invitrogen, Carlsbad, CA, USA). Next, 2 μg of RNA was used for complementary (cDNA) synthesis by using a first strand cDNA kit (TransGen Biotech, Beijing, China), according to the manufacturer's protocol. PCR amplification was carried out in ABI 7300 Thermocycler (Applied Biosystems, Foster City, CA, USA) performed with a SYBR Green PCR kit (Thermo). The PCR cycles were set at 95°C for 10 minutes, followed by 40 cycles at 95°C for 15 seconds, annealing/extension at 60°C for 45 seconds. The primers were designed by Shanghai Sangon Company (Shanghai, China). U6 and β‐actin were used as the internal control. qRT‐PCR data were analysed using 2^−▵▵Ct^ calculation^46^. The primer sequences were shown in Table 1.

**Table 1:** The primer sequences in qRT‐PCR assay

Genes	Primers sequences
miR‐124a	Forward: 5′‐ACACTCCAGCTGGGTAAGGCACGCGGTG‐3′
	Reverse:5′‐CTCACAGTACGTTGGTATCCTTGTGATGTTCGATGCCATATTGTACTGTGAGGGCATTCA‐3′
U6	Forward: 5′‐CTCGCTTCGGCAGCACA‐3′
	Reverse: 5′‐AACGCTTCACGAATTTGCGT‐3′
Pdx‐1	Forward: 5′‐GGATGAAGTCTACCAAAGCTCACGC‐3′
	Reverse: 5′‐CCAGATCTTGATGTGTCTCT‐3′
Pax‐6	Forward: 5′‐AGTTCAGGCCTACCTGATGC‐3′
	Reverse: 5′‐GTCGCGAGTCCCTGTGTC‐3′
Insulin‐1	Forward: 5′‐CCGTCGTGAAGTGGAGGA‐3′
	Reverse: 5′‐CAGTTGGTAGAGGGAGCAGAT‐3′
Ngn3	Forward: 5′‐CATACCTAGGGACTGCTCCGA‐3′
	Reverse: 5′‐CATACAAGCTGTGGTCCGCTA‐3′
GK	Forward: 5′‐AAAGAAGGCAGTTTTGGGGC‐3′
	Reverse: 5′‐CCTGTTCCACCCATCCTTCT‐3′
Nephrin	Forward: 5′‐GAGTTCCTGGGAGAGCAAGT‐3′
	Reverse: 5′‐TCGGGTTACATTCCACAGCT‐3′
podocin	Forward: 5′‐TCCAAAGCCATCCAGTTCCT‐3′
	Reverse: 5′‐TGGCAGCCTCACATCCTTAA‐3′
CD2AP	Forward: 5′‐AGCCCAGGACGATTCAGAAA‐3′
	Reverse: 5′‐TCCGAAGTTTCACAGAGCCT‐3′
caspase‐3	Forward: 5′‐ACTGGACTGTGGCATTGAGA‐3′
	Reverse: 5′‐GCACAAAGCGACTGGATGAA‐3′
Bax	Forward: 5′‐CAGCTCTGAGCAGATCATGAAGACA‐3′
	Reverse: 5′‐GCCCATCTTCTTCCAGATGGTGAGC‐3′
Bcl‐2	Forward: 5′‐ACTTGTGGCCCAGATAGGCACCCAG‐3′
	Reverse: 5′‐CGACTTCGCCGAGATGTCCAGCCAG‐3′
Notch1	Forward: 5′‐AGGTTACACTGGCTCTGCAT‐3′
	Reverse: 5′‐GTTGACATCCATCTCGCACC‐3′
NICD	Forward: 5′‐CCCTTGCTCTGCCTAACGC‐3′
	Reverse: 5′‐GGAGTCCTGGCATCGTTGG‐3′
Hes1	Forward: 5′‐ACACCGGACAAACCAAAGAC‐3′
	Reverse: 5′‐CGCCTGTTCTCCATGATAGG‐3′
Delta	Forward: 5′‐CTATGCGCCACCATCAAGAC‐3′
	Reverse: 5′‐CCTCAGTTGCGTGTAATCCG‐3′
GAPDH	Forward: 5′‐ATGTCGTGGAGTCTACTGGC‐3′
	Reverse: 5′‐TGACCTTGCCCACAGCCTTG‐3′

### 2.11 Proteins isolation and Western blot

Protein in kidney tissues was extracted using RIPA (Solarbio, Beijing, China) and protein in cells was extracted using EpiQuik Whole Cell Extraction Kit (AmyJet Scientific, Wuhan, Hubei, China). Western blot analysis was performed following standard procedures. Protein lysates were separated in 10% sodium dodecyl sulphate‐polyacrylamide gels and electroblotted onto to a polyvinylidene fluoride membrane (Roche Diagnostics, Mannheim, Germany). Next, immunoblotting was carried out using antibody to Pdx‐1 (rabbit anti‐ Pdx‐1 antibody, 1:250; Abcam), Pax‐6 (rabbit anti‐ Pax‐6 antibody, 1:250; Abcam), insulin‐1 (rabbit anti‐ insulin‐1 antibody, 1:250; Abcam), Ngn3 (rabbit anti‐Ngn3 antibody, 1:250; Abcam), GK (rabbit anti‐GK antibody, 1:250; Abcam), nephrin (rabbit anti‐nephrin antibody, 1:400; Abcam), podocin (rabbit anti‐podocin antibody, 1:250; Abcam), CD2AP (rabbit anti‐CD2AP antibody, 1:250; Abcam), caspase‐3 (rabbit anti‐caspase‐3 antibody, 1:400; Beyotime, Shanghai), Bax (rabbit anti‐Bax antibody, 1:400; Beyotime, Shanghai), Bcl‐2 (rabbit anti‐Bcl‐2 antibody, 1:400; Beyotime, Shanghai), Notch1 (rabbit anti‐Notch1 antibody, 1:400; Beyotime, Shanghai), NICD (rabbit anti‐NICD antibody, 1:400; Beyotime, Shanghai), Hes‐1 (rabbit anti‐Hes‐1 antibody, 1:400; Beyotime, Shanghai), Delta (rabbit anti‐Delta antibody, 1:400; Beyotime, Shanghai), and β‐actin (rabbit anti‐β‐actin antibody, 1:800; Beyotime). Protein loading was determined using mouse anti‐β‐actin monoclonal antibody. Lab Works Image Acquisition and Analysis Software (UVP, Upland, CA, USA) were used to quantify band intensities. The analysis was carried out independently three times.

### 2.12 Terminal deoxynucleotidyl transferase‐mediated dUTP nick‐end labelling (TUNEL) assay

Cell apoptosis was detected with In Situ Cell Death Detection Kit (R&D Systems, USA), according to the manufacturer's protocol. Cells (1 × 10^5 ^cells/well) were seeded in 24‐well plates and were treated. Next, cells were first fixed in 4% paraformaldehyde at 4°C for 30 minutes and permeabilized in 0.1% Triton X‐100, and treated with fluorescein‐12‐dUTP. Finally, the fluorescence was detected using fluorescence microscopy (Zeiss Axiovert 100 M, Carl Zeiss, Germany).

### 2.13 Statistical analysis

Data were expressed as mean ± standard deviation (SD). Differences between two groups were determined by Student's *t* test for unpaired variables. However, differences among three or four groups in vitro study were analysed by one‐way ANOVA and subsequent Tukey's test. Probability (*P*) values <.05 was considered as statistically significant.

## 3 RESULTS

### 3.1 Characterization of BMSCs

**Figure 1:**
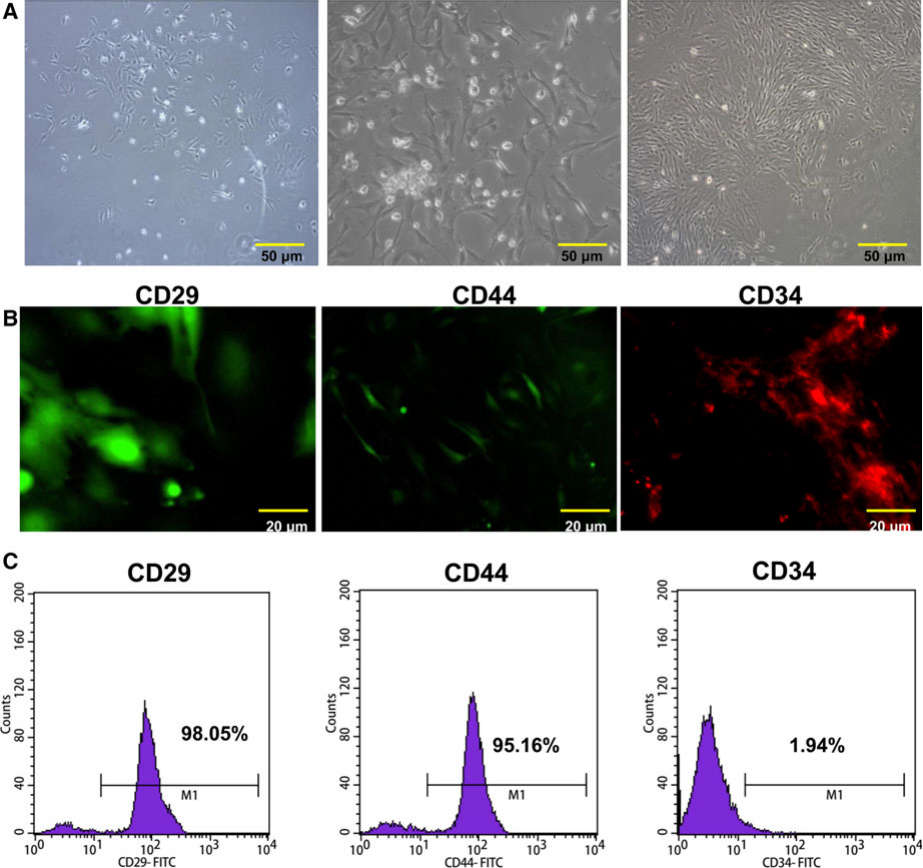
Characterization of BMSCs. A, The cultured BMSCs. Magnification: ×20, Scale bar = 50 μm. B, CD29, CD44 and CD34 in isolated BMSCs were detected by immunofluorescence assay. Magnification: ×40, Scale bar = 20 μm. C, The isolated BMSCs were characterized by a flow cytometer. BMSCs were positive for CD29 and CD44, but negative for CD34

The rat BMSCs presented fibroblast‐like shape. The size of cell increased, and vacuoles and granules appeared in the cells (Figure 1A). Results from immunofluorescence assay showed that the positive expressions of CD29 and CD44, and negative expression of CD34 in BMSCs (Figure 1B). Furthermore, results from flow cytometry showed that the proportions of CD29, CD44 and CD34 were 98.05%, 95.16% and 1.94% in BMSCs, respectively (Figure 1C). Therefore, it was suggested that the isolation of BMSCs was successful.

### 3.2 Differentiation of BMSCs

**Figure 2:**
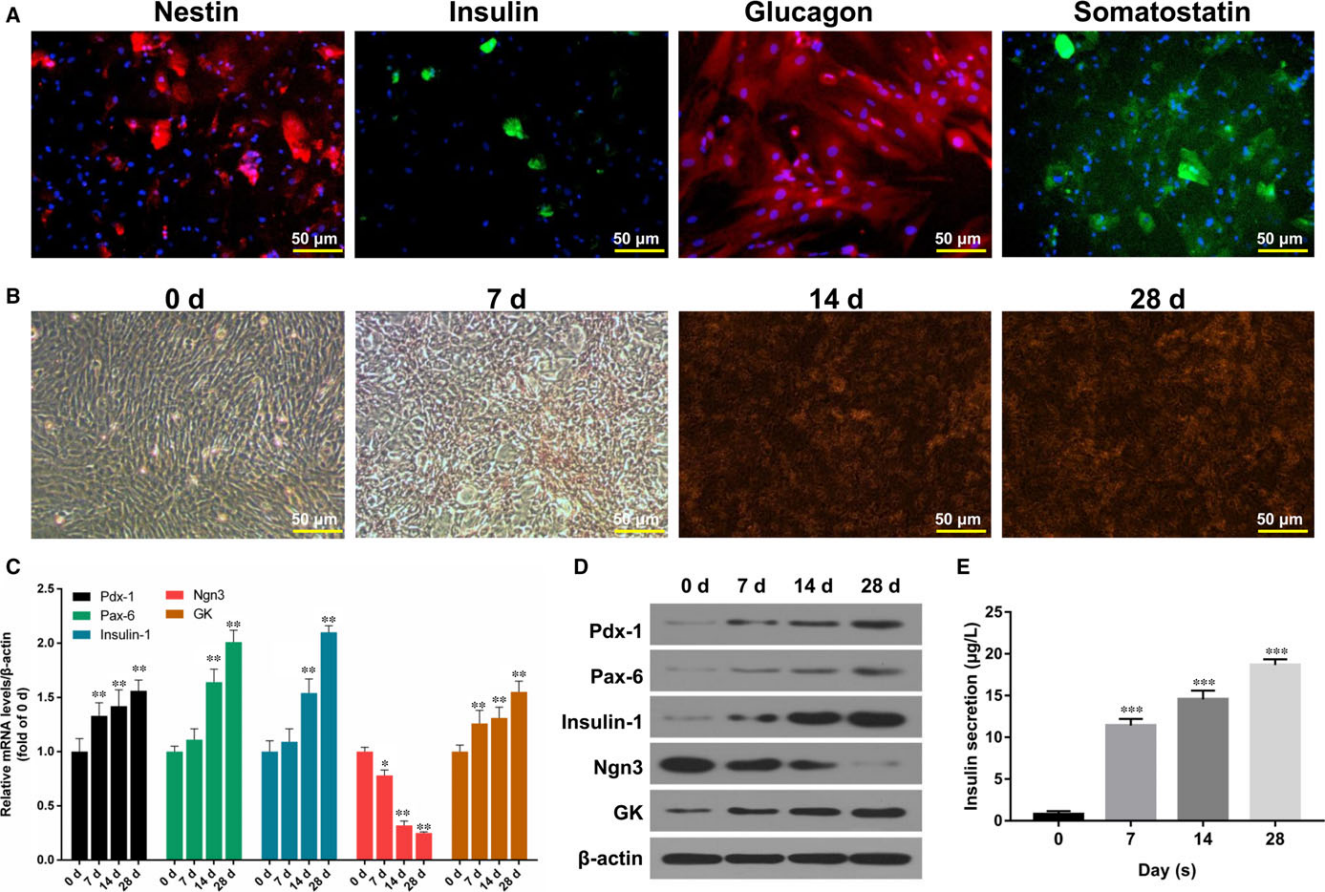
Differentiation of BMSCs. A, Nestin expression was detected by immunofluorescence assay after 24‐h induction; insulin expression was determined by FITC staining after 7 d induction; glucagon expression was analysed Cy3 staining after inducting for 14 d; somatostatin expression was tested by FITC staining after inducting for 14 d. Magnification: ×20, Scale bar = 50 μm. B, The synthesis of insulin was observed by DTZ staining in BMSCs after inducting for 0, 7, 14 and 28 d, respectively. Magnification: ×20, Scale bar = 50 μm. C, The relative mRNA expression levels of Pdx‐1, Pax‐6, Insulin‐1, Ngn3 and GK were determined by qRT‐PCR assay in BMSCs after inducting for 0, 7, 14 and 28 d, respectively (*P < .05 or **P < .01). D, The protein expression levels of Pdx‐1, Pax‐6, Insulin‐1, Ngn3 and GK were analysed by Western blot assay. E, The production of insulin secretion was measured by radioimmunoassay in BMSCs after inducting for 0, 7, 14 and 28 d, respectively (***P < .001)

Our results showed that nestin was highly expressed in BMSCs after 24‐hour induction. Insulin was positive in BMSCs after being induced for 7 days. The expression of glucagon and somatostatin were positive in BMSCs after being induced for 14 days (Figure 2A). In addition, the results of DTZ staining showed that BMSCs turned brown red after being induced for 14 days, suggesting that the cytoplasm of BMSCs contains zinc ion. The above results proved that BMSCs was induced into islet‐like cells (Figure 2B). The expression levels of Pdx‐1, Pax‐6, Insulin‐1, Ngn3 and GK in BMSCs were then determined by qRT‐PCR and Western blot assays. The results indicated that accompanied with the differentiation of BMSCs, Pdx‐1, Pax‐6, Insulin‐1 and GK expressions were gradually increased, and Ngn3 expression was gradually decreased (*P* < .05, *P* < .01, Figure 2C and D). Furthermore, the production of insulin in BMSCs was detected by performing radioimmunoassay. The results indicated that the production of insulin was significantly increased with the differentiation of BMSCs progressed (*P* < .001, Figure 2E).

### 3.3 MiR‐124a promoted insulin secretion and the expression of islet cell‐specific transcription factors in BMSCs

**Figure 3:**
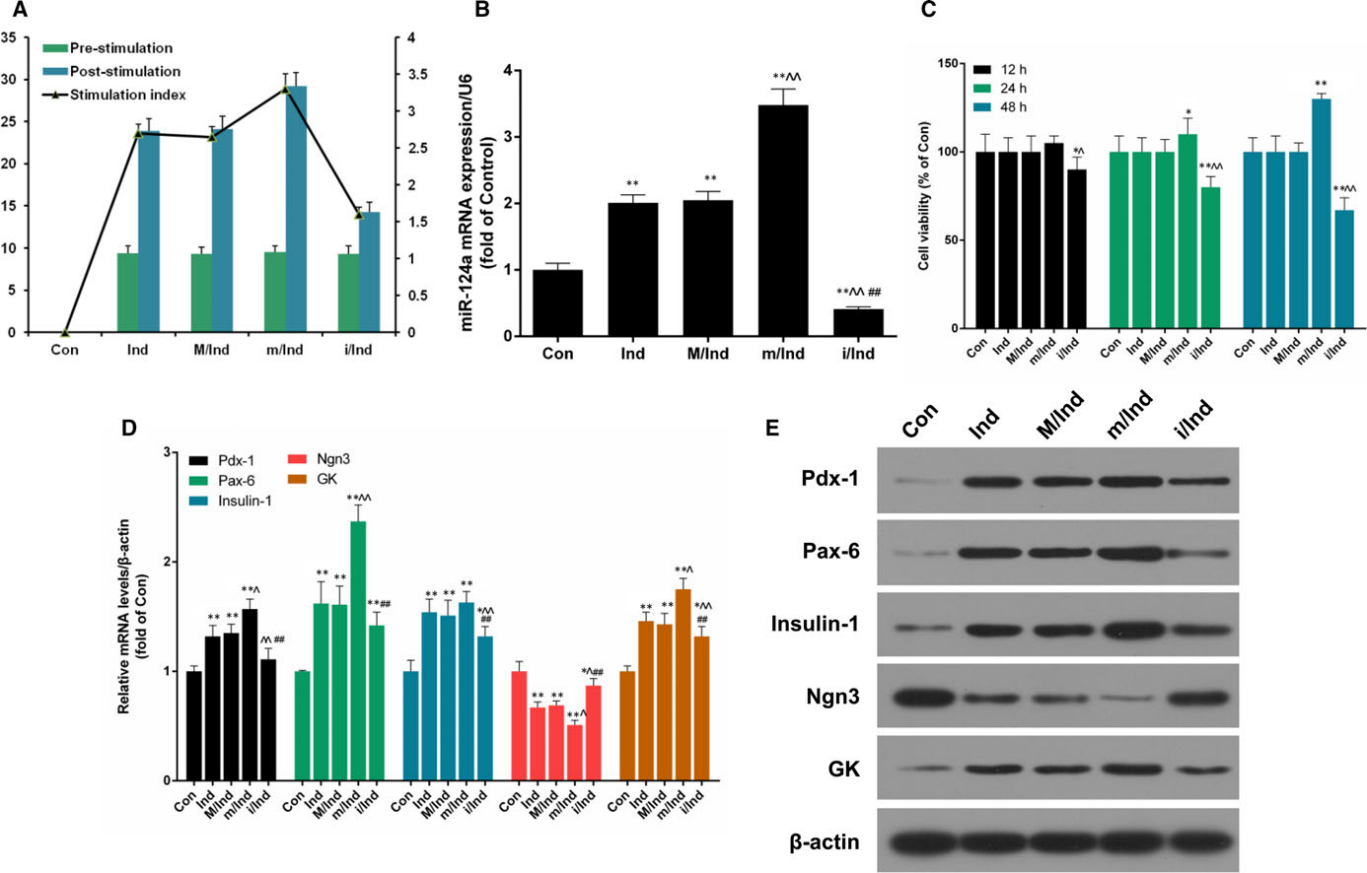
miR‐124a affected insulin secretion, proliferation as well as the expressions of Pdx‐1, Pax‐6, Insulin‐1, Ngn3 and GK in BMSCs. BMSCs were treated with PBS (Con) and 50 mg/L TSPG (Ind) for 28 d, and then the induced‐BMSCs were transfected with mock (M/Ind), miR‐124a mimics (m/Ind) and miR‐124a inhibitors (i/Ind) for 48 h, respectively. A, The production of insulin secretion was detected by radioimmunoassay in treated BMSCs. B, The expression level of miR‐124a was measured by qRT‐PCR assay (**P < .01 vs Con; ^^*P* < .01 vs M/Ind; ## *P* < .01 vs m/Ind). C, Cell viability was detected by CCK‐8 assay in treated BMSCs at 12, 24 and 48 h, respectively (*P < .05, **P < .01 vs Con; ^*P* < .05, ^^*P* < .01 vs M/Ind). D, The mRNA expression levels of Pdx‐1, Pax‐6, Insulin‐1, Ngn3 and GK were determined by qRT‐PCR assay in treated BMSCs (*P < .05, **P < .01 vs Con; ^*P* < .05, ^^*P* < .01 vs M/Ind; ##*P* < .01 vs m/Ind). E, The protein expression levels of Pdx‐1, Pax‐6, Insulin‐1, Ngn3 and GK were detected by Western blot assay in treated BMSCs

To investigate whether miR‐124a play crucial roles in BMSCs or not, BMSCs were treated with PBS (Con) and 50 mg/L TSPG (Ind) for 28 days. The induced‐BMSCs were then transfected respectively with mock (M/Ind), miR‐124a mimics (m/Ind) and miR‐124a inhibitors (i/Ind) for 48 hours. The results indicated that the insulin secretion increased significantly in Ind, M/Ind and m/Ind groups after glucose stimulation, and that the insulin secretion decreased sharply in i/Ind group after glucose stimulation (Figure 3A). In addition, the insulin secretion after induction for 28 days in BMSCs that were transfected with miR‐124a mimics was higher than that in other groups (Figure 3A). Moreover, the expression level of miR‐124a went up significantly after BMSCs were transfected with miR‐124a mimics (*P* < .05, *P* < .01, Figure 3B). CCK‐8 results also showed that the cell viability increased significantly after BMSCs were transfected with miR‐124a mimics, however, it decreased significantly after the BMSCs were transfected with miR‐124a inhibitors for 12, 24 and 48 hours (*P* < .05, *P* < .01, Figure 3C). Furthermore, qRT‐PCR and Western blot assays were used to detect the expression levels of islet cell‐specific transcription factors including Pdx‐1, Pax‐6, Insulin‐1, Ngn3 and GK. The results indicated that the expression levels of Pdx‐1, Pax‐6, insulin‐1 and GK increased significantly in BMSCs after induction for 28 days, and that Pdx‐1, Pax‐6, insulin‐1 and GK expressions increased more sharply in m/Ind group than those in M/Ind group, however, Pdx‐1, Pax‐6, insulin‐1 and GK expressions decreased more significantly in i/Ind group than those in M/Ind group. Simultaneously, the expression of Ngn3 was decreased significantly in BMSCs after 28 days induction. The decrease of Ngn3 was more obvious in m/Ind group than that in M/Ind group. Moreover, Ngn3 expression was increased in i/Ind group than that in M/Ind group (*P* < .05, *P* < .01, Figure 3D and E).

### 3.4 BMSCs in combination with miR‐124a enhanced podocytes cell viability treated with HG

**Figure 4:**
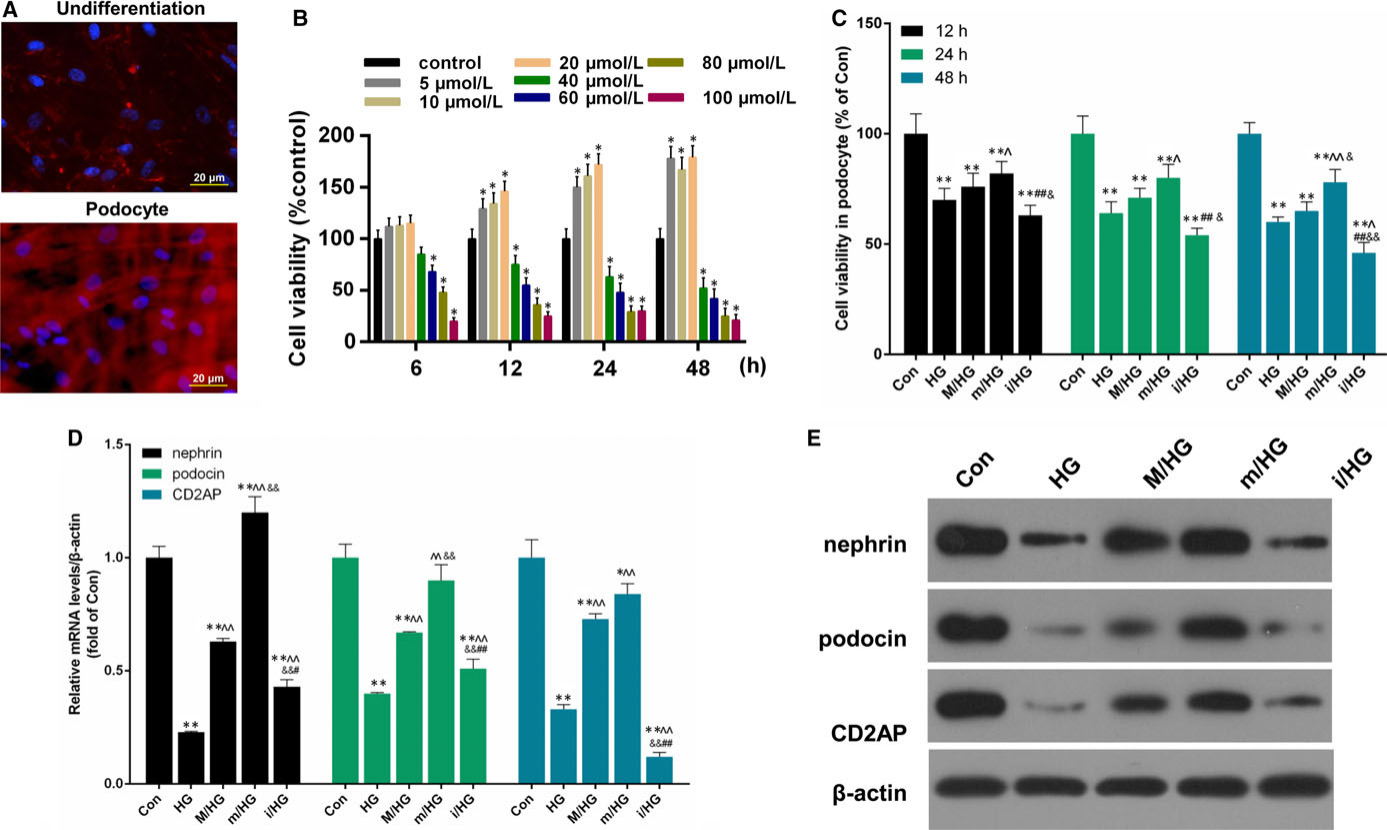
BMSCs combined with miR‐124a enhanced podocytes cell proliferation mediated by HG. A, Podocytes cells were detected by immunofluorescence assay. The cells were visualized by a fluorescence microscope. Magnification: ×40, Scale bar = 20 μm. B, Podocytes cells were treated with PBS (Control) and glucose at 5 mmol/L, 10 mmol/L, 20 mmol/L, 40 mmol/L, 60 mmol/L, 80 mmol/L and 100 mmol/L for 6, 12, 24, 48 h, respectively. CCK‐8 assay was performed to detect the cell viability of podocytes cells. *P < .05 vs Control. C, Podocytes cells were treated with PBS (Con) and 40 mmol/L HG (HG) for 24 h and were then co‐cultured with BMSCs, which were transfected with mock (M/HG), miR‐124a mimics (m/HG) and miR‐124a inhibitors (i/HG) for 48 h, respectively. CCK‐8 assay was used to measure the cell viability ability of podocytes cells. **P < .01 vs Con; ^*P* < .05 or ^^*P* < .01 m/HG vs M/HG; ##*P* < .01 i/HG vs M/HG; &*P* < .05 or &&*P* < .01 i/HG vs m/HG. D, The mRNA expression levels of Nephrin, podocin and CD2AP were measured by qRT‐PCR assay in treated podocytes cells. **P < .01 vs Con; ^*P* < .05 or ^^*P* < .01 m/HG vs M/HG; ##*P* < .01 i/HG vs M/HG; &*P* < .05 or &&*P* < .01 i/HG vs m/HG. E, The protein expression levels of Nephrin, podocin and CD2AP were detected by Western blot assay in treated podocytes cells

To further examine the biological significance of BMSCs in combination with miR‐124a on podocytes cell proliferation treated with high‐glucose (HG), immunofluorescence assay was first used to identify the characteristics of podocytes cells. The results indicated that synaptopodin protein highly expressed in podocytes (Figure 4A). Then, Podocytes were treated with PBS (Control) and glucose at 5 mmol/L, 10 mmol/L, 20 mmol/L, 40 mmol/L, 60 mmol/L, 80 mmol/L and 100 mmol/L for 6, 12, 24, 48 hours, respectively. CCK‐8 results showed that low concentration Glucose enhanced the viability of podocytes cells in dose‐dependent and time‐dependent manners. However, HG dampened the cell viability (Figure 4B). Subsequently, podocytes were treated with PBS (Con) and 40 mmol/L HG (HG) for 24 hours and were then co‐cultured with BMSCs, which were respectively transfected with mock (M/HG), miR‐124a mimics (m/HG) and miR‐124a inhibitors (i/HG) for 48 hours. CCK‐8 results indicated that the cell viability decreased in podocytes treated with high‐glucose. After co‐culturing with BMSC that was transfected with miR‐124a mimics, the cell viability increased significantly compared with M/HG groups. After co‐culturing with BMSC that was transfected with miR‐124a inhibitors, the cell viability decreased further, compared with M/HG groups (*P* < .05, *P* < .01, Figure 4C). In addition, we also found that HG significantly inhibited the expression levels of nephrin, podocin and CD2AP in podocytes, and that the pre‐treatment of BMSC transfection with miR‐124a mimics significantly increased nephrin, podocin and CD2AP expressions compared with M/HG groups. However, the pre‐treatment of BMSC transfection with miR‐124a inhibitors significantly decreased nephrin, podocin and CD2AP expressions in comparison with M/HG groups (*P* < .05, *P* < .01, Figure 4D and E).

### 3.5 BMSCs in combination with miR‐124a inhibited podocytes cell apoptosis treated with HG

**Figure 5:**
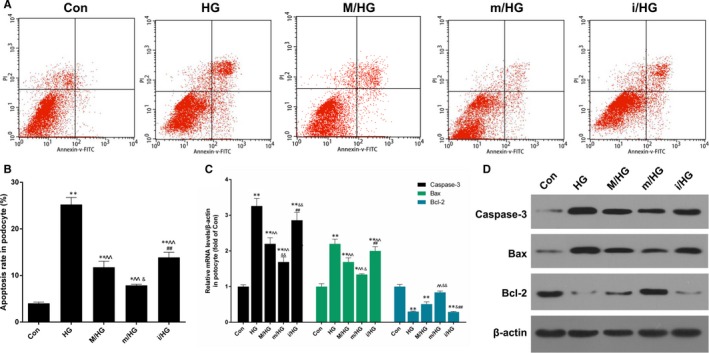
BMSCs in combination with miR‐124a inhibited podocytes cell apoptosis mediated by HG. Podocytes cells were treated with PBS (Con) and 40 mmol/L HG (HG) for 24 h and were then co‐cultured with BMSCs, which were transfected with mock (M/HG), miR‐124a mimics (m/HG) and miR‐124a inhibitors (i/HG) for 48 h, respectively. A, Apoptosis ability was detected by flow cytometry. B, The number of apoptosis was respectively quantitatively analysed (*P < .05 or **P < .01 vs Con; ^^*P* < .01 vs HG; ##*P* < .01 i/HG vs M/HG; &*P* < .05 m/HG vs M/HG). C, The mRNA expression levels of apoptosis‐related genes (caspase‐3, Bax and Bcl‐2) were measured by qRT‐PCR assay, respectively (**P < .01 vs Con; ^^*P* < .01 vs HG; &*P* < .05 or &&*P* < .01 vs M/HG; ##*P* < .01 i/HG vs m/HG). D, The protein expression levels of caspase‐3, Bax and Bcl‐2 were detected by Western blot assay

Furthermore, we explored the effects of BMSCs in combination with miR‐124a on the apoptosis of podocytes cells treated with HG. The results showed that HG significantly promoted apoptosis of podocytes cells, and that the pre‐treatment of BMSC transfected with miR‐124a mimics sharply inhibited apoptosis compared with M/HG groups. The pre‐treatment of BMSC transfected with miR‐124a inhibitors was observed to significantly enhance apoptosis in comparison with M/HG groups (*P* < .05, *P* < .01, Figure 5A and B). In addition, we also found that HG noticeably up‐regulated caspase‐3 and Bax expressions, and down‐regulated Bcl‐2 expression in podocytes, and that the pre‐treatment of BMSC transfection with miR‐124a mimics decreased significantly caspase‐3 and Bax expressions, and increased Bcl‐2 expression in comparison with M/HG group. The pre‐treatment of BMSC transfection with miR‐124a inhibitors was found to largely increase caspase‐3 and Bax expressions and decreased Bcl‐2 expression, compared with M/HG group (*P* < .05, *P* < .01, Figure 5C and D).

### 3.6 BMSCs combined with miR‐124a regulated the expressions of Notch1, NICD, Hes1 and Delta

**Figure 6:**
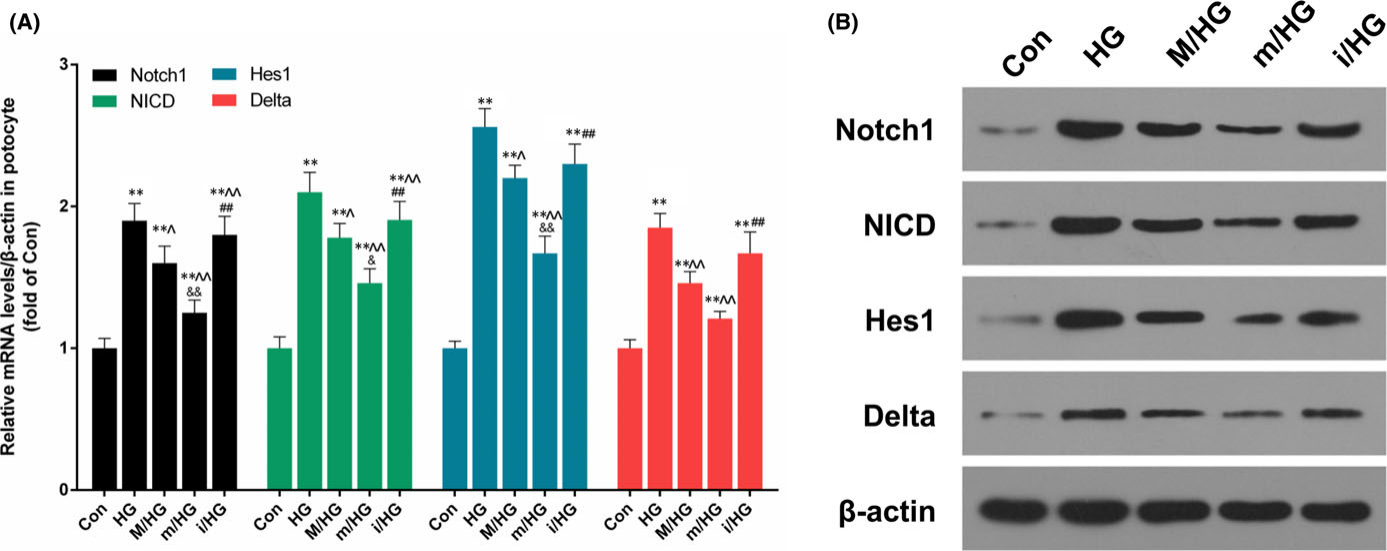
BMSCs in combination with miR‐124a regulated Notch1, NICD, Hes1 and Delta expressions. Podocytes cells were treated with PBS (Con) and 40 mmol/L HG (HG) for 24 h and were then co‐cultured with BMSCs, which were transfected with mock (M/HG), miR‐124a mimics (m/HG) and miR‐124a inhibitors (i/HG) for 48 h, respectively. A, The mRNA expression levels of Notch1, NICD, Hes1 and Delta were measured by qRT‐PCR assay, respectively (**P < .01 vs Con; ^*P* < .05 or ^^*P* < .01 vs HG; &*P* < .05 or &&*P* < .01 m/HG vs M/HG; ##*P* < .01 i/HG vs M/HG). B, The protein expression levels of Notch1, NICD, Hes1 and Delta were detected by Western blot assay

We further demonstrated the effect of BMSCs combined with miR‐124a on Notch signalling pathway. Our results showed that HG significantly up‐regulated the expressions of Notch1, NICD, Hes1 and Delta in podocytes, and that the pre‐treatment of BMSC transfection with miR‐124a mimics noticeably decreased Notch1, NICD, Hes1 and Delta expressions compared with M/HG group. However, the pre‐treatment of BMSC transfection with miR‐124a inhibitors significantly increased the expressions of Notch1, NICD, Hes1 and Delta in comparison with M/HG group (*P* < .05, *P* < .01, Figure 6A and B).

### 3.7 BMSCs combined with miR‐124a reduced kidney injury in DN rats

**Figure 7:**
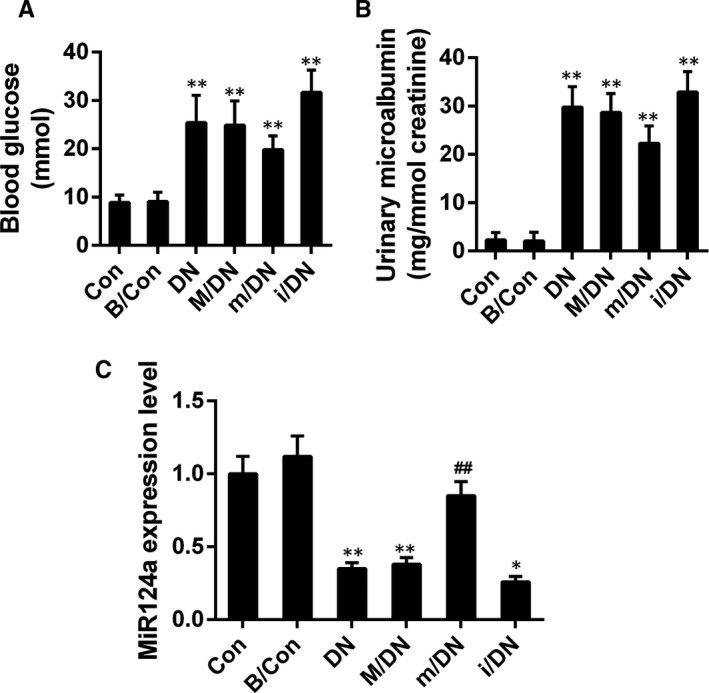
BMSCs in combination with miR‐124a decreased the level of urinary albumin excretion and blood glucose. SD rats were divided into control group (Con), BMSCs group (B/Con, 3 × 10^6^ BMSCs), diabetic model group (DN), mock (M/DN), miR‐124a mimics (m/DN) and miR‐124a inhibitors (i/DN). A, The level of UAM was assessed by biochemical analyser; (**P < .01 vs B/Con). B, The blood glucose was determined by glucose cytometer; (**P < .01 vs B/Con). C, The expression of miR124a in each group was analysed by qRT‐PCR assay; (**P < .01 vs B/Con); ##*P* < .01 vs. DN

We found that the bodyweights of DN rats were increased significantly in DN model group, compared to those of Con and B/Con groups. However, bodyweights of rats treated with BMSCs transfection with miR‐124a mimics were decreased compared to DN group. The 24‐hours urinary albumin excretion (UAE) was increased significantly in DN groups, indicating that the renal function was recovered by the treatment of BMSCs in combination with miR‐124a, although there was no significant difference in terms of kidney index (UMA/Ucr)^47^ between m/DN and the model group (*P* > .05, Figure 7A). Compared to control and B/Con groups, blood glucose level higher than 16.7 mmol/L were observed in DN and M/DN groups. Whereas blood glucose level was decreased in m/DN group compared to DN and M/DN groups (*P* > .05, Figure 7B). The expression of miR124a was shown in Figure 7C. According to our results, DN models were successfully established by HFD/STZ treatment, and the model could be used in further experimental study. Our data showed that the overexpression of miR‐124a could decrease the weight of DN rats and blood glucose level.

### 3.8 BMSCs in combination with miR‐124a protect kidney tissue from damage

**Figure 8:**
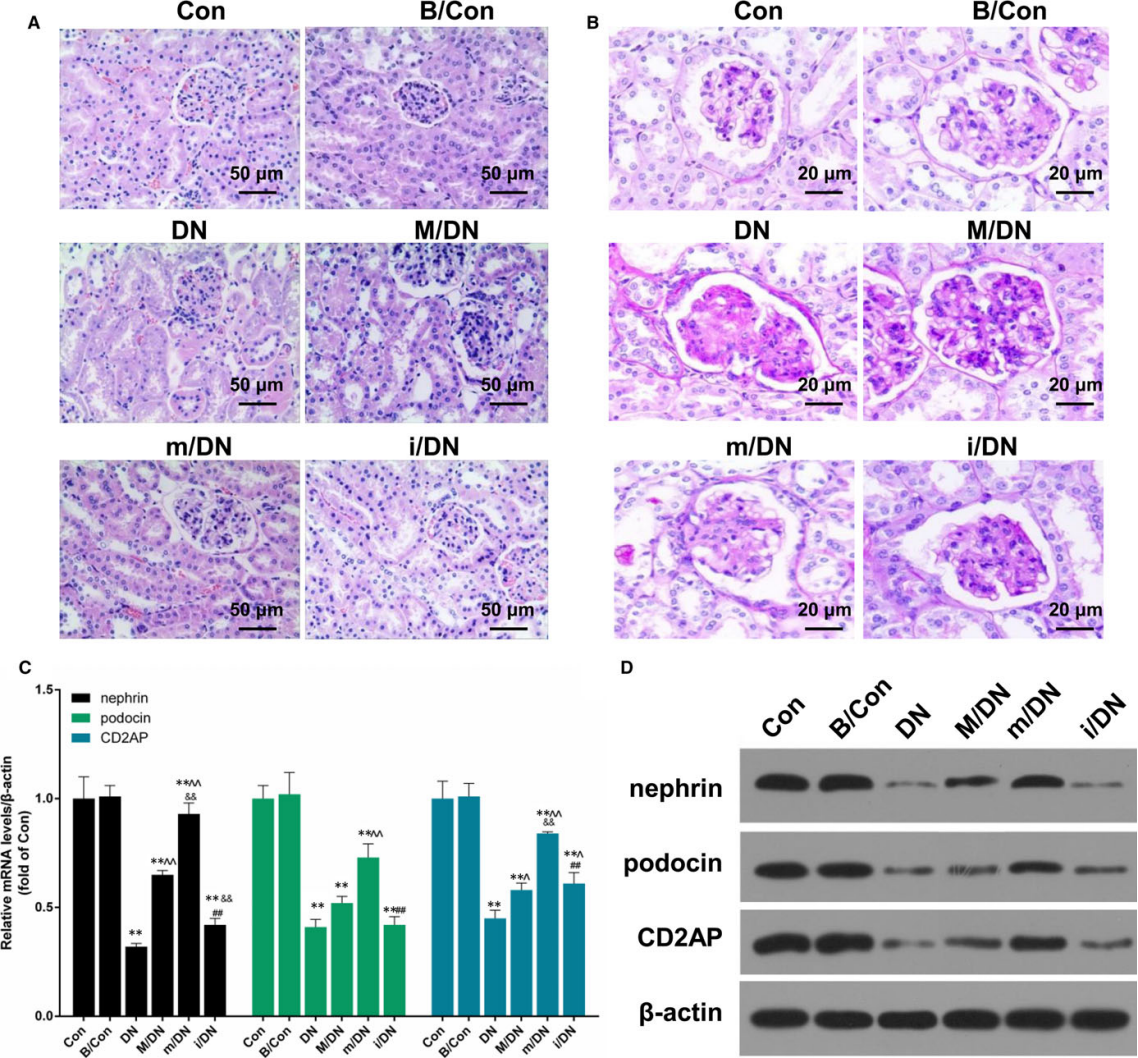
BMSCs in combination with miR‐124a protect kidney tissue from impairment. SD rats were divided into control group (Con), BMSCs group (B/Con, 3 × 10^6^ BMSCs), diabetic model group (DN), mock (M/DN), miR‐124a mimics (m/DN) and miR‐124a inhibitors (i/DN). A, The morphology of rat kidney was observed by HE staining in different groups. B, The extent of glomerular injury was observed by PAS staining in different groups. C, The mRNA expression levels of nephrin, podocin, and CD2AP were analysed by qRT‐PCR assay (**P < .01 vs B/Con; ^*P* < .05 or ^^*P* < .01 vs DN; &&*P* < .01 m/DN vs M/DN; ##*P* < .01 i/DN vs M/DN). D, The protein expression levels of nephrin, podocin and CD2AP were detected by Western blot assay

**Figure 9:**
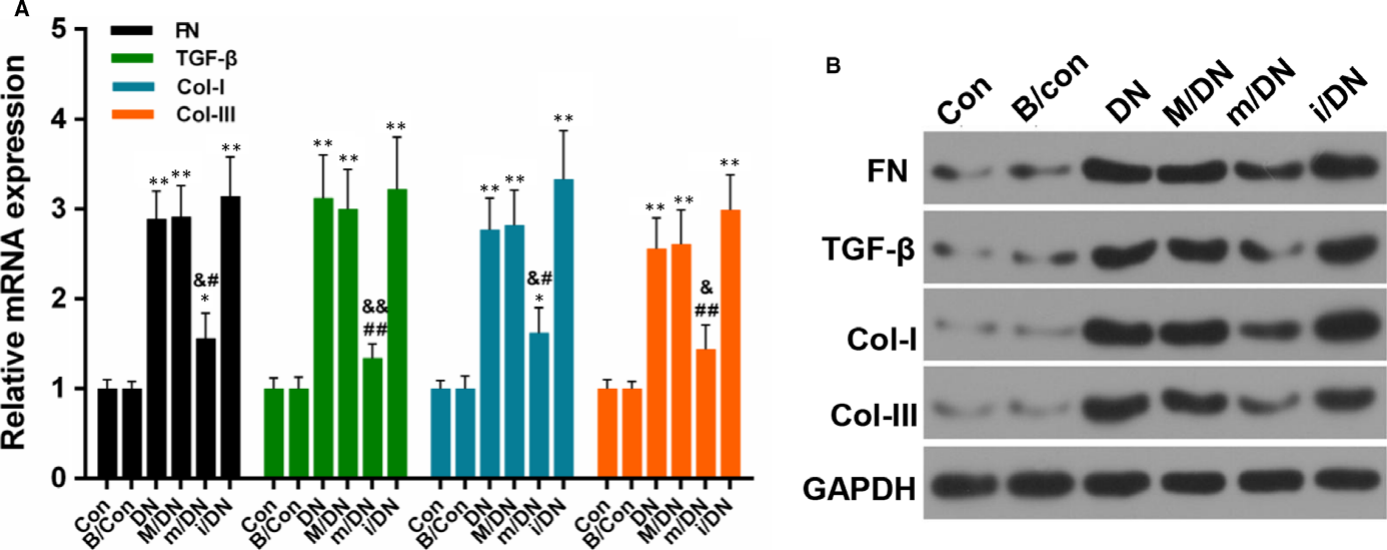
BMSCs combined with miR‐124a protect against kidney fibrosis. SD rats were divided into control group (Con), BMSCs group (B/Con, 3 × 10^6^ BMSCs), diabetic model group (DN), mock (M/DN), miR‐124a mimics (m/DN) and miR‐124a inhibitors (i/DN). A, The mRNA expression levels of FN, TGF‐β, Col‐I and Col‐III. (*P < .05 or **P < .01 vs B/Con; ^*P* < .05 or ^^*P* < .01 vs DN; &*P* < .05 or &&*P* < .01 m/DN vs M/DN; #*P* < .05 or ##*P* < .01 i/DN vs M/DN). B, The protein expression levels of FN, TGF‐β, Col‐I and Col‐III were detected by Western blot assay

To confirm the effect of BMSCs transfection with miR‐124a mimics/inhibitors against DN rats, SD rats were divided into control group (Con), BMSCs group (B/Con, 3 × 10^6^ BMSCs), diabetic model group (DN), mock (M/DN), miR‐124a mimics (m/DN) and miR‐124a inhibitors (i/DN). The renal pathological changes were observed by HE and PAS staining. The HE staining results indicated that compared with Con and B/Con groups, kidney tissue in DN group appeared glomerular sclerosis, mesangial cells and matrix hyperplasia as well as vacuolar degeneration in epithelia of renal tubules. The PAS staining results observed that the area of glomerular mesangium matrix increased significantly (Figure 8A and B). After treating with BMSCs in DN rats, the extent of pathological injury of kidney injury was markedly alleviated. The effect of BMSCs transfection with miR‐124a mimics was more productive than that with miR‐124a inhibitors/negative control. In addition, we found that the expression levels of nephrin, podocin and CD2AP were lower in DN group than those in Con and B/Con groups, and that nephrin, podocin and CD2AP expressions increased significantly in m/DN group in comparison with DN group (*P* < .05, *P* < .01, Figure 8C and D). Furthermore, we also detected the expression of fibrotic related proteins^48^. The result showed that compared to DN group, the expression of fibronectin (FN), TGF‐β1, Col‐I, and Col‐III was depressed by the treatment of BMSCs in combination with miR‐124a. By contrast, the co‐treatment of BMSCs and miR‐124a inhibitors enhanced the expression of FN, TGF‐β1, Col‐I, and Col‐III compared to DN group (Figure 9A and B).

### 3.9 BMSCs in combination with miR‐124a inhibited nephrocyte apoptosis

**Figure 10:**
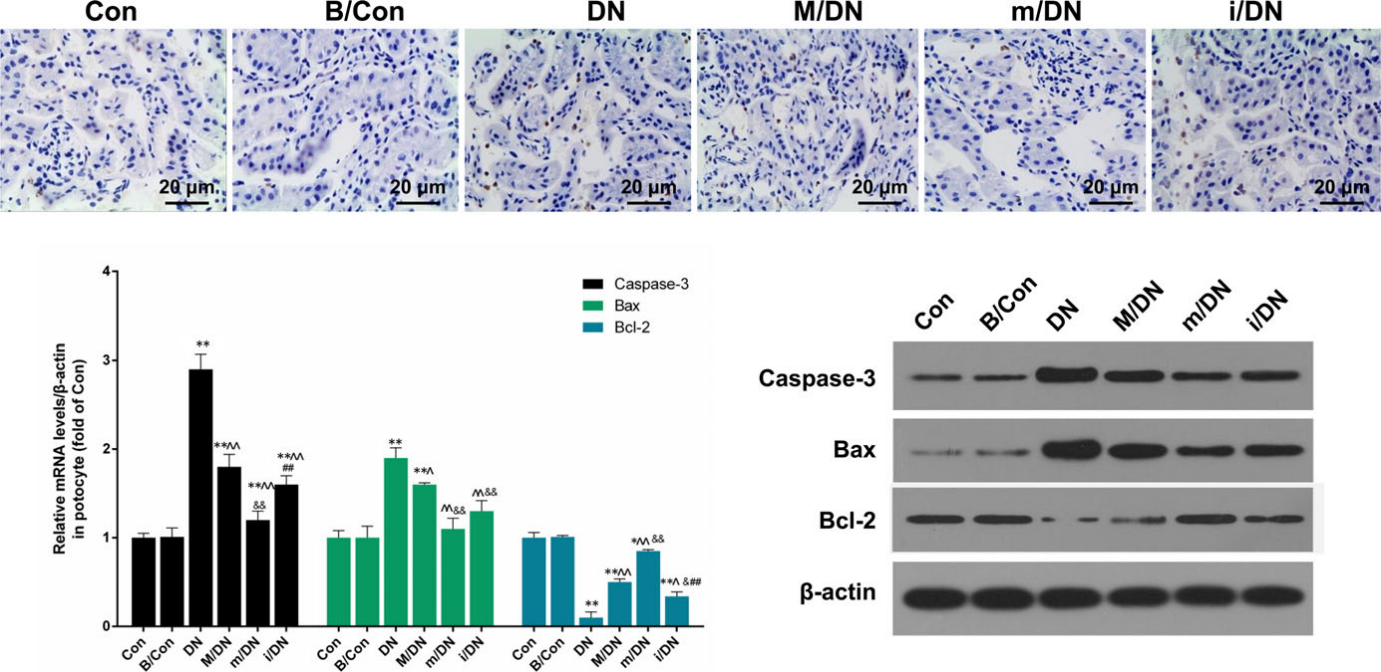
BMSCs in combination with miR‐124a inhibited nephrocyte apoptosis. SD rats were divided into Con, B/Con, DN, M/DN, m/DN and i/DN. A, The apoptosis ability was detected by TUNE L assay. Magnification: ×40, Scale bar = 20 μm. B, The mRNA expression levels of caspase‐3, Bax and Bcl‐2 were analysed by qRT‐PCR assay (**P < .01 vs B/Con; ^*P* < .05 or ^^*P* < .01 vs DN; &*P* < .05 or &&*P* < .01 m/DN vs M/DN; ##*P* < .01 i/DN vs M/DN). C, The protein expression levels of n caspase‐3, Bax and Bcl‐2 were detected by Western blot assay

The method of TUNEL was used to detect apoptosis in SD rats that were divided into Con, B/Con, DN, M/DN, m/DN and i/DN groups. We found that the apoptosis rate was higher in DN group than that in Con and B/Con groups (Figure 10A). Cell apoptosis was increased significantly in DN group. But the apoptosis was decreased in m/DN group compared to DN group. The apoptosis of i/DN group was increased compared to that of the DN group (Figure 10A). In addition, apoptosis‐related genes including caspase‐3, Bax and Bcl‐2 were detected by qRT‐PCR and Western blot assays with an aim to further explore the mechanism. Our results showed that the expression of pro‐apoptosis factors (caspase‐3 and Bax) was significantly increased, whereas the expression of anti‐apoptosis factor (Bcl‐2) was decreased noticeably in DN group. However, the presence of BMSCs transfected with miR‐124a mimics decreased the expression of caspase‐3 and Bax and increased the expression of Bcl‐2. The presence of BMSCs transfected with miR‐124a inhibitors exerted the effects that opposite to BMSCs transfected with miR‐124a mimics (*P* < .05, *P* < .01, Figure 10B and C).

## 4 DISCUSSION

Diabetes mellitus (DM) has a high morbidity rate and is usually accompanied with complications that seriously threats human health^49^, ^50^. The main treatments for DM are oral hypoglycaemic medications and/or insulin. However, some severe medication side effects emerge from the treatment^51^. In recent years, many studies have proved that the application of hematopoietic stem cell or islet transplantation was effective method in treating type 1 diabetes^52^, ^53^. Although this treatment could produce certain effects, the long‐term use of immunosuppressive agents could produce side effects for the patients of islet transplant^54^, ^55^. Thus, it is necessary to seek for a method that is safe and long‐acting for treating diabetes and its complications.

Studies have shown that type 1 diabetes mellitus (T1DM) and type 2 diabetes mellitus (T2DM) are closely related to the immune and inflammatory response or damage^56^, ^57^. Therefore, an effective inhibition or blocking of the immune injury can protect β cells from damage, maintaining the efficacy of pancreas islet function and correct insulin resistance^58^. Under certain conditions, mesenchymal stem cells (MSC) can differentiate into pancreas islet β‐like cells^59^. MSCs have functions such as tissue repairing, angiogenesis, antioxidant effects, metabolic regulation and insulin resistance^60^. Thus, MSCs play an important role in the pathogenesis of multiple aspects of diabetes.

Our findings indicated that most BMSCs attained fibroblast‐like shape, and can express the surface markers of BMSCs antigens, for example, CD29 and CD44^61^. These results were consistent with the standards of the International Society for Cell Therapy^62^.

In recipient mice, bone marrow transplantation can generate islet cells, which can produce genetic markers and insulin of β‐cells^63^. A previous research also has shown that BMSCs can differentiate into insulin‐expressing cells under controllable conditions in vitro^64^. In our study, we characterized BMSCs and detected the differentiation of BMSCs. We found that BMSCs can not only express neural stem cells marker, nestin, but also pancreas‐associated genes including insulin, glucagon and somatostatin. Therefore, our results indicated that BMSCs could be induced into islet‐like cells. It has been reported that transcripts for proinsulin I and II such as Pax 4, Ngn‐3, Isl 1, Pdx‐1, Pax‐6, Insulin‐1 and glucokinase (GK) participate in the signal‐mediated secretion of insulin in pancreatic β cells^65^. Our results also revealed that with the differentiation of BMSCs, the expressions of Pdx‐1, Pax‐6, Insulin‐1 and GK were increased gradually, while the expression of Ngn3 was decreased. In addition, we found that the production of insulin was significantly increased with the differentiation of BMSCs.

MicroRNAs (miRNAs) are short (about 18‐22 nucleotides) non‐coding RNAs that predominantly regulate target mRNAs by binding to the 3′‐untranslated region (UTR), causing degradation or translation inhibition^66^, ^67^. Increasing evidence indicates that miRNAs modulate tumour initiation and progression such as proliferation, invasion and metastasis^68^, ^69^. Recently, miR‐124a was regarded as a tumour suppressor in various human cancers^70^. A previous research has shown that miRNA‐124a was abnormally expressed in type 2 diabetic human pancreatic islets^21^. In our study, we found that miR‐124a increased the insulin secretion and promoted the cell proliferation of Podocytes significantly. Simultaneously, miR‐124a down‐regulated Ngn3 expression, while it up‐regulated the expression levels of Pdx‐1, Pax‐6, insulin‐1 and GK.

Podocytes are terminally differentiated visceral epithelial cells that located outside the glomerular capillaries^71^. In diabetic nephropathy, podocytes injury leads to the disruption of the filtration barrier and protein leakage. The loss of podocytes has been indicated as an important early pathologic marker^72^. Research has shown that high glucose can induce podocyte injury^73^. In our study, we found that HG inhibited the proliferation ability of podocytes cells, and that BMSCs in combination with miR‐124a promoted proliferation, and inhibited apoptosis of podocytes cells treated with HG. We also found that HG significantly inhibited the expressions of nephrin, podocin and CD2AP in podocytes, and that BMSCs in combination with miR‐124a significantly up‐regulated the expressions of nephrin, podocin and CD2AP. HG was observed to up‐regulate significantly the expressions of caspase‐3 and Bax, and down‐regulated Bcl‐2 expression in podocytes. Moreover, BMSCs in combination with miR‐124a inhibited caspase‐3 and Bax expressions and promoted Bcl‐2 expression.

Notch pathway plays important roles in embryonic development, cellular proliferation and differentiation^34^. Notch pathway was relevant to glomerular disease including DN, focal segmental glomerulosclerosis and hydropigenous nephritis as well as glomerular sclerosis^74^. This pathway consists of four receptors, which are Notch1‐Notch4 and five ligands: Jagged1, Jagged2, Delta‐like (Dll) 1, Dll3 and Dll4^75^. The binding of ligand and Notch receptor induces a conformational change, which allows the γ‐secretase‐mediated protease to release the Notch intracellular domain (NICD)^76^. NICD travels into the nucleus in which it activates the transcription of downstream genes such as Hes1 and Hey1 genes, and affects cellular differentiation, proliferation and apoptosis^77^. In our study, we demonstrated that HG significantly up‐regulated the expressions of Notch1, NICD, Hes1 and Delta in podocytes, and that BMSCs in combination with miR‐124a inhibited the expressions of Notch1, NICD, Hes1 and Delta. Thus, those data suggested that BMSCs and miR‐124a inhibited Notch signalling pathway.

Nephrin is a transmembrane protein in the podocyte that belongs to a member of the immunoglobulin superfamily^78^. Nephrin can induce phosphorylation of several target protein and reduce the apoptosis of podocytes^79^. Podocin connects nephrin by CD2AP. This produces an enhanced effect on nephrin signal transduction^80^. Abnormal expressions of these factors may affect the glomerular filtration barrier permeability, resulting in the generation of proteinuria^80^. Nephrin and CD2AP play a key role in maintaining glomerular filtration barrier integrity. In this study, we established a DN model, and found that BMSCs in combination with miR‐124a significantly increased the expressions of nephrin, podocin and CD2AP. High glucose can stimulate fibroblast collagen synthesis and the expression of transforming growth factor‐1 (TGF‐β1). TGF‐β1 is able to promote collagen synthesis and inhibit collagenase release. Under normal conditions, a negative feedback mechanism exist to maintain the renal balance^81^. However, this mechanism would be disrupted under HG conditions. Our results showed that the BMSCs in combination with miR‐124a inhibited the expressions of FN, TGF‐β1, Col‐I and Col‐III, suggesting that BMSCs in combination with miR‐124a depressed the progression of renal fibrosis. With the fibrosis of the kidney, renal failure is also related to the progressive deletion of renal cells^82^. We found that BMSCs in combination with miR‐124a inhibited nephrocyte apoptosis by regulating the expressions of Bcl‐2, caspase‐3 and Bax.

Collectively, miR124a helped BMSCs differentiate into islet‐like cells and the insulin secretion of BMSCs. Therefore, the depressed renal injury in DN may be explained by that the insulin conferred by BMSCs in the presence of miR124a. In addition, as a safe and effective delivery vehicle in exosomal trafficking, MSCs has been used to deliver miRNA to targeted cells to treat diabetes^83^. Thus, it was also possible that the presence of MSC elevated the transfection efficiency of miR124a and then enhanced the effect of miR124a.

However, one limitation of this study was that there was discrepancy among changes in renal function and in molecular biology. This may due to the fact that the final organ function may affect by not only the molecules we detected, but also some other unknown signals or the cell context. In addition, the precise regulatory mechanisms via which MSCs combined with miR‐124a exerted was not clear in this study. Thus, it will be intriguing to illustrate the regulatory signal pathways involved in this study model, and it will enrich our knowledge on the molecular progression of DN.

## 5 CONCLUSION

In summary, our studies have successfully identified and isolated BMSCs. We proved that miR‐124a promoted insulin secretion, proliferation and affected the expressions of Pdx‐1, Pax‐6, Insulin‐1, Ngn3 and GK in BMSCs. Our results also indicated that MSCs in combination with miR‐124a enhanced proliferation and inhibited apoptosis of podocytes cells mediated by HG. In addition, we observed that BMSCs in combination with miR‐124a inhibited Notch signalling pathway in podocytes cells. We have demonstrated that BMSCs in combination with miR‐124a protected kidney tissue from impairment and inhibited nephrocyte apoptosis in vivo.

## CONFLICT OF INTEREST

The authors declare no conflict of interest.
